# Solitary but Sinister: A Pulmonary Nodule Detected Through Lung Cancer Screening Unveils Small Cell Lung Cancer

**DOI:** 10.7759/cureus.106891

**Published:** 2026-04-12

**Authors:** Tirth D Chauhan, Mukesh K Ahluwalia, William Porter

**Affiliations:** 1 Pulmonology and Critical Care, Saint James School of Medicine, Chicago, USA; 2 Department of Pulmonary and Critical Care Medicine, Respiratory, Critical Care, and Sleep Specialists, Chicago, USA; 3 Department of Pathology, Northwest Community Hospital, Chicago, USA

**Keywords:** histopathology (hp), immunohistochemistry (ihc), lung biopsy, lung cancer screening (lcs), robotic-assisted bronchoscopy (rab), small cell lung cancer (sclc), solitary pulmonary nodule, surgical case reports, very-limited stage small cell lung cancer

## Abstract

Small cell lung cancer (SCLC) is an aggressive malignancy that typically presents as a centrally located mass with early metastatic spread and only rarely manifests as a solitary pulmonary nodule (SPN) without lymphadenopathy. We report a 63-year-old active smoker whose low-dose computed tomography (LDCT) screening identified a 1.6 cm solitary nodule in the right lower lobe. Positron emission tomography (PET) confirmed metabolic activity without evidence of metastasis, although the nodule grew to 2.2 cm over six months. Robotic-assisted bronchoscopy with endobronchial ultrasound-guided biopsy revealed a high-grade neuroendocrine carcinoma. Immunohistochemical staining confirmed SCLC (TTF-1 positive, synaptophysin positive, p40 negative, Napsin A negative, and Ki-67 >80%). The tumor was staged as IA (T1c N0 M0), and the patient was considered a candidate for curative surgical resection. However, she succumbed to severe cardiovascular comorbidities prior to intervention. This case illustrates the “solitary but sinister” presentation of SCLC as a SPN and underscores the importance of maintaining a high index of suspicion, prompt tissue diagnosis, and expedited management in high-risk patients to avoid missing a narrow window for potentially curative treatment.

## Introduction

Small cell lung cancer (SCLC) is an aggressive high-grade neuroendocrine malignancy typically characterized by its predilection for central airway involvement, rapid doubling time, and early systemic dissemination [1,2]. Although it accounts for approximately 15% of all lung cancer diagnoses, its presentation as a solitary pulmonary nodule (SPN) without lymphadenopathy is an exceptionally rare clinical entity and has been reported only in a small minority of cases [2,3]. The detection of SPNs has increased significantly with the widespread adoption of low-dose computed tomography (LDCT) for lung cancer screening. Large, randomized screening trials, including the National Lung Screening Trial (NLST) and the NELSON trial, have demonstrated that pulmonary nodules are identified in approximately 25-40% of screened individuals, the majority of which are benign or represent early-stage non-small cell lung cancer (NSCLC), particularly adenocarcinoma [4,5,6]. The clinical significance of SPNs lies in the diagnostic challenge they present; accurately distinguishing an early-stage, potentially curable malignancy from a benign lesion is critical for determining the appropriate clinical pathway and avoiding unnecessary invasive procedures.

By contrast, SCLC is typically associated with bulky mediastinal lymphadenopathy and locally advanced disease at presentation [1,7]. As a result, a small peripheral SCLC lesion may closely mimic the radiographic appearance of early-stage adenocarcinoma, especially when initial size and morphology are considered in isolation, posing a significant diagnostic pitfall and potential delay in definitive diagnosis [2,8]. Despite the rising detection of SPNs, there remains a significant knowledge gap regarding the clinical recognition and management of SCLC when it masquerades as a peripheral nodule. Because peripheral SCLC is frequently presumed to be NSCLC, this diagnostic bias can lead to critical delays in definitive tissue diagnosis and treatment. We report a case of very-limited-stage SCLC presenting as an SPN detected through routine lung cancer screening.

## Case presentation

A 63-year-old African American woman with a medical history of asthma, obstructive sleep apnea, hypertension, diabetes mellitus, and heart failure with reduced ejection fraction presented to the pulmonary clinic for lung cancer screening. She was an active smoker (one to two cigarettes daily) with an extensive smoking history. Physical examination was unremarkable, and she reported no hemoptysis, weight loss, or other constitutional symptoms.

**Figure 1:**
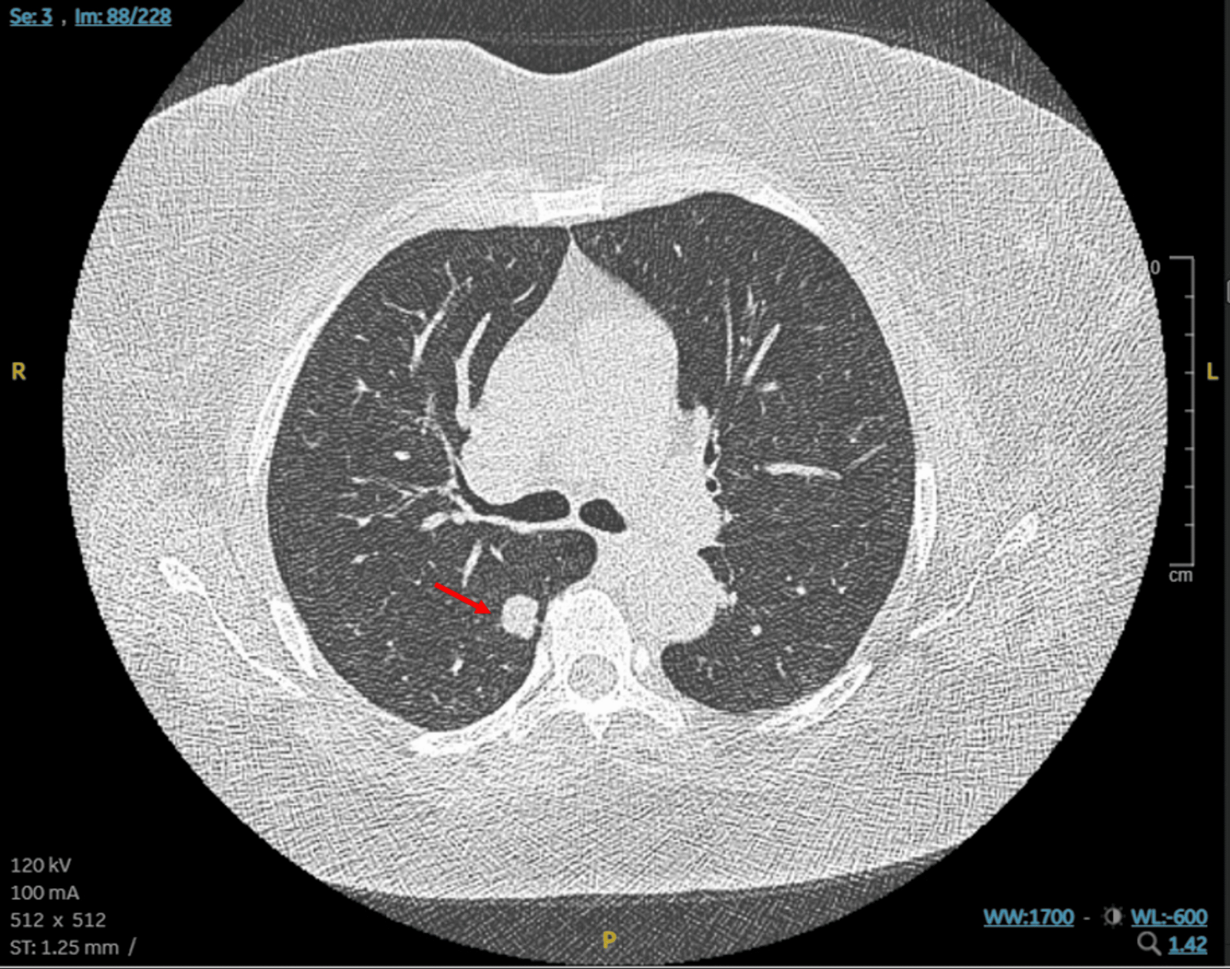
Low-dose computed tomography scan (LDCT) of the chest. Low-dose CT (LDCT) of the chest (axial view) demonstrating a 1.6 cm solitary pulmonary nodule (red arrow) in the superior segment of the right lower lobe. Note the complete absence of associated central airway involvement or hilar/mediastinal lymphadenopathy, which is an atypical presentation for small cell lung cancer.

**Figure 2:**
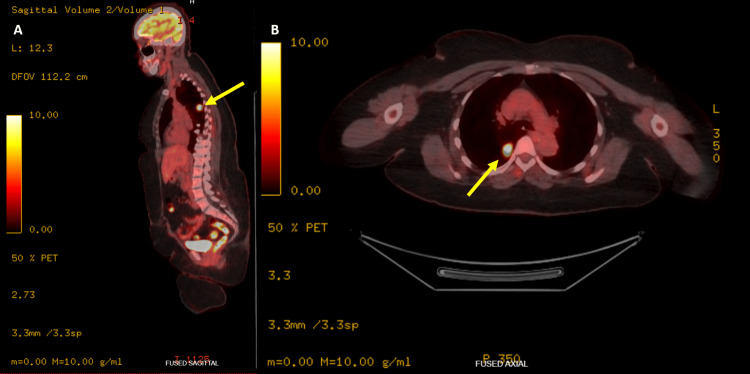
Positron emission tomography (PET) scan. F-18 FDG PET/CT scan (sagittal and axial views) showing intense FDG uptake tightly localized to the right lower lobe nodule (yellow arrows). The hypermetabolic activity strongly suggested malignancy, while the absence of regional nodal or distant metastasis confirmed very-limited-stage disease.

In May, a routine screening LDCT revealed a 1.6 cm solid nodule in the superior segment of the right lower lobe, with no significant hilar or mediastinal lymphadenopathy (Figure 1). Further diagnostic evaluation was delayed by four months due to patient's travel. A subsequent positron emission tomography (PET) scan in late September demonstrated intense fluorodeoxyglucose uptake confined entirely to the right lower lobe nodule without evidence of distant metastasis or locoregional nodal involvement (Figure 2).

**Figure 3:**
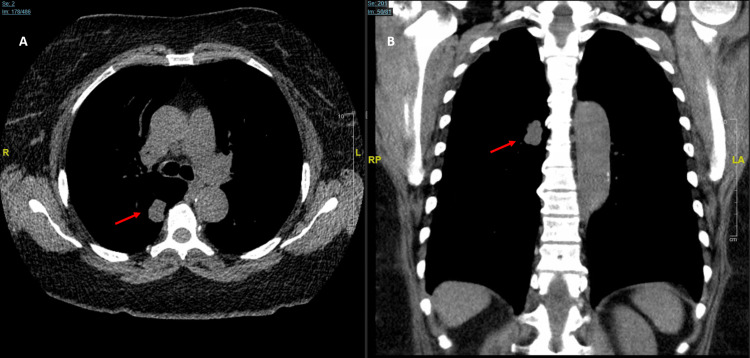
Interval CT chest (axial and coronal views) on the ion robotic protocol obtained prior to bronchoscopy. CT chest (axial and coronal views) obtained prior to robotic bronchoscopy; the images demonstrate significant interval growth of the right lower lobe mass (red arrows) from 1.6 cm to 2.2 x 1.4 cm over a six-month period, highlighting the rapid tumor kinetics characteristic of high-grade neuroendocrine carcinoma.

In early November, following optimization of her heart failure, the patient underwent a robotic-navigational bronchoscopy with endobronchial ultrasound. Interval CT imaging at the time of the procedure demonstrated growth of the lesion to 2.2 x 1.4 cm (Figure 3). This rapid interval growth over six months in an active smoker with a heavy smoking history significantly elevated clinical suspicion for an aggressive malignancy, such as a rapidly progressing adenocarcinoma or a high-grade neuroendocrine tumor. Given this aggressive tumor kinetic profile, coupled with the lesion's high metabolic activity on the prior PET scan, prompt definitive tissue diagnosis via biopsy was strictly prioritized over continued radiographic surveillance to avoid stage migration and preserve a potential window for early intervention. Transbronchial biopsies, needle aspiration, and bronchoalveolar lavage of the right lower lobe lesion were performed, alongside biopsies of lymph node stations 11R, 7, and 11L.

**Figure 4:**
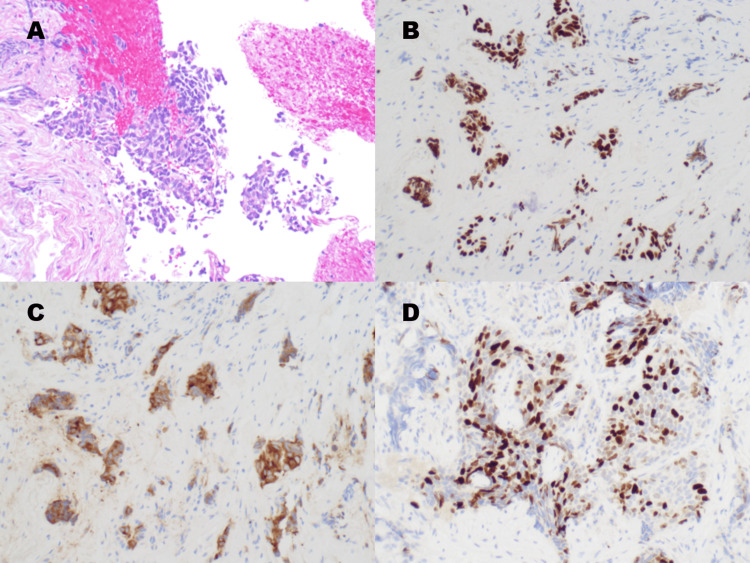
Histopathology and Immunohistochemical evaluation. (A) Hematoxylin and eosin (H&E) stain demonstrating infiltration by small round blue cells with hyperchromatic nuclei, scant cytoplasm, and characteristic crush artifact. (B) Immunohistochemical staining for TTF-1 showing diffuse nuclear positivity, supporting primary lung origin. (C) Synaptophysin staining demonstrating strong cytoplasmic positivity, confirming neuroendocrine differentiation. (D) Ki-67 proliferation marker showing a high index (>80%), consistent with high-grade malignancy and distinguishing the tumor from typical carcinoid.

Cytopathological evaluation (hematoxylin and eosin stain) revealed nests of small round blue cells with hyperchromatic nuclei, scant cytoplasm, and prominent crush artifact (Figure 4A). These cytologic features were consistent with high-grade neuroendocrine carcinoma vs. a poorly differentiated adenocarcinoma. Crush artifact is a classic artifact often seen in small cell carcinoma biopsies due to the fragility of the cell nuclei.

Immunohistochemical staining showed strong, diffuse positivity for TTF-1 and synaptophysin, and focal weak positivity for chromogranin (Figure 4B, 4C). The significance of TTF-1 is that it is a marker primarily for lung and thyroid tissues. In the context of a lung nodule, a positive result strongly suggests a primary lung origin for the tumor. Approximately 85-90% of small cell lung carcinomas are TTF-1 positive. Synaptophysin is a neuroendocrine marker, and the tumor’s strong cytoplasmic positivity for synaptophysin confirms its neuroendocrine differentiation. The tumor was also negative for p40 and Napsin A, effectively ruling out squamous cell carcinoma and adenocarcinoma. The Ki-67 proliferative index was markedly elevated at >80-90% (Figure 4D). Ki-67 stain marks cells that are actively proliferating. The proliferation index of >80-90% indicates a very aggressive, high-grade tumor. Carcinoid tumors, both typical and atypical, generally have a lower Ki-67 index (often <20-30%). Small cell carcinomas and large cell neuroendocrine carcinomas typically have very high Ki-67 indices (often 80-100%). After combining the findings of cytopathologic and immunohistochemical evaluation, a diagnosis of high-grade SCLC was confirmed.

Given the tumor size (<3 cm) and absence of nodal metastasis, the patient was diagnosed with limited-stage SCLC, clinically staged as IA (T1c N0 M0). As per current National Comprehensive Cancer Network guidelines for very-limited-stage SCLC, she was deemed a candidate for curative surgical resection [1]. Unfortunately, prior to surgical intervention, the patient succumbed to complications related to her severe cardiovascular and respiratory comorbidities.

## Discussion

SCLC accounts for approximately 13-15% of all lung cancers and classically presents as a centrally located mass with mediastinal lymphadenopathy [1]. By contrast, presentation as an SPN without nodal involvement is exceptionally rare, comprising less than 5% of SCLC cases [2]. While this presentation is well documented, it remains infrequently encountered in clinical practice and is diagnostically challenging. Peripheral nodules often lack the definitive radiographic features of SCLC, further contributing to diagnostic uncertainty and complicating early recognition [8]. Large screening trials have demonstrated that while SPNs are frequently detected, the overwhelming majority represent NSCLC, particularly adenocarcinoma, with SCLC being underrepresented in peripheral lesions [4,5]. Therefore, a diagnostic bias exists in which peripheral nodules are often presumed to represent NSCLC until proven otherwise.

The diagnostic journey in our case highlights the limitations of relying on radiographic appearance alone and underscores the necessity of analyzing tumor kinetics. SCLC is characterized by a remarkably rapid volume doubling time (VDT), typically reported in the literature to be between 30 and 80 days, making it one of the most rapidly proliferating solid tumors [9,10]. In sharp contrast, typical early-stage peripheral adenocarcinomas, which this lesion initially mimicked, often exhibit VDTs exceeding 150 to 400 days [11,12]. In our patient, the nodule's maximum diameter progressed from 1.6 cm to 2.2 cm over a six-month (approximately 180 days) interval. When analyzing this cross-sectional increase volumetrically, it represents a substantial and rapid tumor expansion. While peripheral SCLCs can occasionally exhibit slightly more erratic or slower growth kinetics than classic central SCLC masses, this interval growth pace significantly exceeds the expected behavior of a benign inflammatory lesion or a low-grade adenocarcinoma. This quantitative deviation from typical NSCLC kinetics is a hallmark that should immediately trigger suspicion for a high-grade neuroendocrine pathology. Although the patient remained node-negative during this interval, the aggressive baseline kinetics of these tumors demonstrate the danger of standard six- to 12-month radiographic surveillance protocols in this context. It reinforces the urgency of obtaining an expedited tissue biopsy in high-risk patients when an indeterminate nodule demonstrates rapid early expansion.

Definitive diagnosis of these peripheral lesions relies on robust histopathological and molecular profiling. Earlier studies suggested that up to two-thirds of peripheral SCLC cases exhibit combined histology rather than pure small cell features [3]. However, advances in genomic profiling have refined our understanding of these tumors, emphasizing that even pure SCLC possesses distinct molecular characteristics that differentiate it from other pulmonary neuroendocrine tumors [7,13]. According to the 2021 World Health Organization classification, immunohistochemistry remains the gold standard for differentiation, relying heavily on markers such as TTF-1, synaptophysin, and Ki-67 [14]. In our case, tumor cells were strongly positive for TTF-1 and synaptophysin, confirming lung lineage and neuroendocrine differentiation, while testing negative for p40 and Napsin A, which effectively ruled out squamous cell carcinoma and adenocarcinoma. Crucially, the Ki-67 proliferative index was markedly elevated at greater than 80-90%. This high proliferative rate is a hallmark of SCLC, effectively distinguishing this lesion from typical or atypical carcinoid tumors, which generally present with much lower Ki-67 indices (<30%) and require a significantly different, less aggressive management approach [15].

The expansion of lung cancer screening programs has shifted the stage distribution at diagnosis, particularly for NSCLC [4]. However, emerging evidence suggests that low-dose computed tomography (LDCT) screening may be less effective in downstaging SCLC due to its rapid tumor kinetics and central origin [6]. Our case represents a rare but clinically significant scenario in which screening enabled the detection of a peripheral SCLC that was potentially curable with surgical resection. Based on tumor size (<3 cm) and absence of nodal or distant metastasis, the disease was clinically staged as IA (T1c N0 M0) [16]. While standard treatment for SCLC typically involves systemic chemotherapy with or without radiation due to early dissemination, current National Comprehensive Cancer Network guidelines and recent surgical reviews support surgical resection, specifically lobectomy, with mediastinal lymph node dissection, followed by adjuvant chemotherapy for T1-2, N0 disease [1,17]. However, for patients with significant physiological frailty or prohibitive operative risk, Stereotactic Body Radiation Therapy (SBRT) has emerged as a highly effective alternative. Recent literature, including a 2024 review of limited-stage SCLC management, suggests that SBRT can achieve excellent local control with lower morbidity than conventional radiation or surgery in the high-risk population [18]. In the context of our patient, whose severe heart failure made surgical candidacy precarious, SBRT would have represented a therapeutic alternative had she reached the treatment phase. This case highlights a critical pivot point in management; had the nodule been dismissed as inflammatory or monitored less aggressively, the window for surgical cure would likely have closed. Tragically, while this patient was a candidate for curative-intent surgery, her significant comorbidities led to her demise before intervention could occur, yet the case firmly validates the necessity of lung cancer screening and reinforces the need for expedited evaluation to uncover these solitary but sinister lesions.

## Conclusions

Our case underscores several critical clinical takeaways for the management of SPNs in high-risk patients: 1) Analyze tumor kinetics: Peripheral pulmonary nodules should not be universally presumed to represent NSCLC. The rapid progression of this nodule from 1.6 cm to 2.2 cm over six months reflects a growth rate consistent with SCLC’s rapid volume doubling time (30-80 days). This quantitative "red flag" should trigger immediate suspicion for high-grade neuroendocrine pathology. 2) Prioritize biopsy over surveillance: Rapid interval growth of an SPN demands immediate clinical attention and expedited tissue biopsy rather than continued radiographic surveillance. As seen in the aggressive biology of SCLC, standard monitoring intervals can lead to rapid stage migration and the closure of a curative window. 3) Rely on comprehensive pathology: Definitive diagnosis of peripheral neuroendocrine lesions requires robust immunohistochemistry (e.g., TTF-1, synaptophysin, and Ki-67). A Ki-67 index exceeding 80-90% is essential to differentiate high-grade SCLC from less aggressive carcinoid tumors, which require vastly different management strategies. 4) Recognize the full curative spectrum: When SCLC is identified early at the solitary nodule stage, the treatment paradigm shifts toward curative intent. While surgical lobectomy is the standard for fit patients, SBRT represents a vital curative alternative for patients with prohibitive operative risks, such as the severe heart failure observed in this case.
